# Clinical benefit of locoregional therapy for oligometastasis after surgery for pancreatic cancer: a single center retrospective study

**DOI:** 10.1186/s12885-026-16566-2

**Published:** 2026-07-20

**Authors:** Koki Ichio, Takahiro Einama, Mayuko Ohara, Kazuki Kobayashi, Naoto Yonamine, Takazumi Tsunenari, Yasuhiro Takihata, Mikiya Takao, Hideki Ueno, Yoji Kishi

**Affiliations:** Department of Surgery, National Defense Medical College, 3-2 Namiki, Tokorozawa, Saitama 359-8513 Japan

**Keywords:** Pancreatic Neoplasms, Oligometastasis, Local Neoplasm Recurrence, Combined Modality Therapy, Survival Analysis

## Abstract

**Background:**

The prognosis of patients with pancreatic cancer (PC) is generally poor. However, favorable outcomes have recently been reported in selected patients with metastatic PC who receive multidisciplinary treatment. Oligometastasis (OM) represents a limited metastatic state and may be associated with better prognosis than multiple metastases (MM). The clinical role of locoregional therapy (LT) for postoperative OM remains unclear.

**Methods:**

We included all consecutive patients with pancreatic malignancies who underwent macroscopically curative resection between 2013 and 2018. Patients with postoperative recurrence were divided into OM and MM groups. Survival outcomes and treatment after recurrence were compared between the groups.

**Results:**

In total, 150 patients were evaluated. Postoperative recurrence was classified as OM in 29 patients and MM in 71 patients. Among the patients with OM, 10 underwent LT. Overall survival and survival after recurrence were significantly longer in the OM group than in the MM group. Among patients with OM, those who underwent LT showed longer survival after recurrence than those who received chemotherapy alone.

**Conclusions:**

Patients with postoperative OM after pancreatic cancer surgery showed more favorable survival outcomes than those with MM. In carefully selected patients with postoperative OM, LT combined with chemotherapy was associated with longer survival after recurrence. However, because of the retrospective single-center design, small number of treated patients, and potential selection bias, LT should be regarded as a possible treatment option rather than an established therapeutic strategy.

## Background

Pancreatic cancer (PC) is globally representative of cancers associated with a poor prognosis [1, 2]. However, with the development of multidisciplinary treatment [3], favorable outcomes have been increasingly reported even in patients with metastatic PC [4–6]. Hellman and Weichselbaum proposed the term “oligometastasis” (OM), defined as a state of metastatic lesions usually limited in number to less than five without rapid spread to other sites [7]. Reports of OM in patients with PC being associated with favorable outcomes have been increasing recently [8–12]. In contrast, the prognosis of patients with OM after surgery for PC and effect of locoregional treatment (LT) including surgical resection or radiotherapy have rarely been evaluated except for in case reports [13] or a limited number of case series [14, 15]. In addition, there might be publication bias whereby only patients who showed favorable outcomes following LT were reported. Therefore, the actual prognosis of patients who received LT for OM as postoperative recurrence is unclear.

In this study, we compared survival rates between patients with OM and non-OM (multiple metastases [MM]) after primary surgery for PC and evaluated the efficacy of LT, including surgical resection and radiotherapy, for OM.

## Materials and methods

### Patients

We included all consecutive patients with pancreatic malignancies who underwent macroscopically curative resection at the National Defense Medical College Hospital between January 2013 and December 2018. Patients who underwent non-curative or palliative surgery, those without resection, and those with non-malignant pancreatic disease were not included. This study period was selected to ensure sufficient follow-up for evaluating postoperative recurrence, treatment after recurrence, including chemotherapy and LT, and survival after recurrence. Patients who underwent surgery after 2018 were not included because their survival outcomes after recurrence were considered potentially immature for the present analysis.

### Surgical procedure and postoperative surveillance

Operative methods were determined based on the tumor location. All patients underwent regional lymph node dissection. The pathologic stage was assessed according to the TNM classification system of the Union for International Cancer Control (UICC), 8th edition [16]. Adjuvant chemotherapy was initiated as soon as possible after recovery of the patient’s physical condition. The first-choice regimen for adjuvant chemotherapy was S-1 monotherapy for 6 months [17]. In patients for whom adverse events such as diarrhea were a concern, gemcitabine monotherapy was selected. After surgery, each patient was followed up at our outpatient clinics. Tumor markers were evaluated every three months and computed tomography (CT) was performed every six months or when recurrence was suspected for 5 years after surgery. We diagnosed recurrence basically by CT, and fluorodeoxyglucose positron emission tomography (PET/CT) or magnetic resonance imaging (MRI) was also conducted to aid with the diagnosis.

### The definitions of OM and MM

In this study, OM after surgery was defined as a limited number (one to five) of metastatic lesions at any sites and/or lymph nodes. MM was defined as six or more metastatic lesions at one single site or metastases to two or more sites or organs. Metastases were detected by imaging studies.

### Criteria for locoregional therapy

The indication for LT for postoperative OM was determined through multidisciplinary team discussions. LT was considered for patients with lesions judged to be clinically controllable and technically treatable, without rapid systemic progression. In principle, systemic chemotherapy was administered before LT to assess disease behavior. Disease control was defined as the absence of apparent enlargement of known recurrent lesions and the absence of newly developed metastatic lesions during chemotherapy. When clinically feasible, we aimed to confirm disease control for approximately 6 months before considering LT. The treatment modality was selected according to the location of recurrence, expected extent of resection, technical feasibility, and the patient’s general condition. Surgical resection was considered when complete removal was expected to be feasible with acceptable invasiveness. Radiotherapy was selected when surgical resection was considered technically demanding, required an extensive procedure, or was unsuitable because of the patient’s physical condition.

### Statistical analyses

JMP pro version 17.1.0 (SAS Institute, Cary, NC, USA) was used for all statistical analyses. The Kaplan–Meier test was used to analyze the survival, which was calculated from the date of primary radical resection of PC or postoperative recurrence to death or the last follow-up. They were compared by the log-rank test. Significance was set at a P-value < 0.05.

## Results

### Patient characteristics

**Fig. 1:**
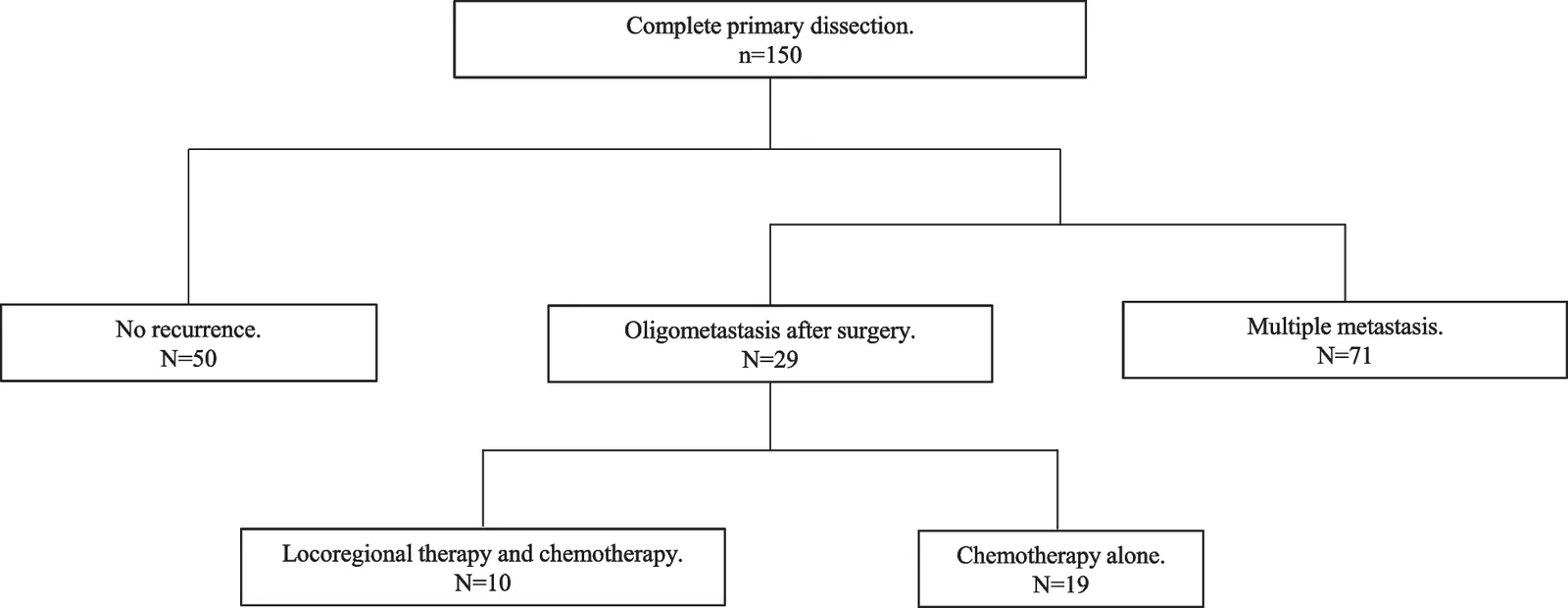
Overview of the study population

In total, 150 PC patients who received initial surgery during the study period were selected. The median age was 71 years (range: 37 to 92), including 88 males and 62 females. The characteristics of patients are indicated in Table 1, and types of treatment for postoperative recurrence are summarized in Fig. 1. After primary resection, 50 patients (33%) had no recurrence, 71 patients (47%) showed MM, and 29 patients (19%) showed OM after surgery. Among patients with non-R0 resection, two developed local recurrence as the initial recurrent pattern and were classified into the OM group, whereas the remaining patients with non-R0 resection developed MM.

**Table 1:** Patient characteristics in this study

Patient number	150
Neoadjuvant chemotherapy^*a^	27 (18.0)
Tumor marker^*b^	
CEA	2.6 (0.6—45.2)
CA19-9	138.3 (0.4—15,270)
DUPAN-2	110 (25—1600)
SPAN-1	48 (3–954)
R0 excision rate^*a^	110 (85.3)
Pathology^*b^	
IDC	112 (74.7)
Invasive IPMC	36 (24)
ACC	1 (0.7)
Invasive MCC	1 (0.7)
Adjuvant chemotherapy	92 (61.3)
T stage^*a^	
Tis	1 (0.7)
T1	10 (6.7)
T2	93 (62.0)
T3	41 (27.3)
T4	5 (0.3)
N stage^*a^	
N0	30 (20.0)
N1	65 (43.3)
N2	55 (36.7)
TNM stage^*a^	
0	1 (0.1)
IA	7 (4.7)
IB	14 (9.3)
IIA	8 (5.3)
IIB	60 (40.0)
III	60 (40.0)

*IDC* Invasive ductal carcinoma, *IPMC* Intraductal papillary mucinous carcinoma, *ACC* Acinar cell carcinoma, *MCC* Mucinous cystadenocarcinoma

^*a^Values are number of patients and percent

^*b^Values are median (range)

The pathologic diagnosis was invasive ductal adenocarcinoma (IDC) in 112 patients, invasive intraductal papillary mucinous carcinoma (inv. IPMC) in 36 patients, acinar cell carcinoma (ACC) in 1 patient, and invasive mucinous cystadenocarcinoma (MCC) in 1 patient. The incidence of OM and MM in each pathology type is shown in Table 2.

**Table 2:** The incidence of OM and MM in each pathology type

	N	No recurrence (%)	Oligometastasis after surgery (%) (Number of patients who underwent locoregional therapy)	Multiple metastases (%)
Total	150	50 (33)	29 (19) (10)	71 (47)
IDC	112	39 (35)	20 (18) (6)	53 (47)
Invasive IPMC	36	10 (28)	8 (22) (3)	18 (50)
ACC	1	0	1 (100) (1)	0
Invasive MCC	1	1 (100)	0	0

*IDC* Invasive ductal carcinoma, *IPMC* Intraductal papillary mucinous carcinoma, *ACC* Acinar cell carcinoma, *MCC* Mucinous cystadenocarcinoma

### Details of locoregional therapy

LT was performed in 10 among 29 patients with OM. The pathology type of the 10 patients was IDC in 6 patients, inv. IPMC in 3 patients, and ACC in one patient. Seven of these patients received adjuvant chemotherapy (S-1) after the operation. Nine of the 10 patients received both chemotherapy and LT, and one patient did not because she refused to receive chemotherapy. LT for 6 patients was administered after confirming its effectiveness. Details of LT are summarized in Table 3.

**Table 3:** Clinical characteristics and outcomes of locoregional therapy

Pathology	Age	Sex	RFS (months)	Pre-LT chemotherapy regimen (months)	Adjuvant Chemotherapy	Site	CA19-9 (U/mL)	Treatment	SAR (months)	Alive/Dead
IDC	71	M	6.0	GnP (7)	S-1	local	21.8	Chemo + Radi	26.0	Alive
inv. IPMC	72	F	16.0	GnP (10)	S-1	Para Ao LN	15.2	Chemo + Radi	56.0	Alive
IDC	81	M	24.0	-	S-1	Lung	5.8	Surgery → Chemo	16.0	Alive
inv. IPMC	62	F	17.0	GnP (10)	S-1	Para Ao LN → Para Ao LN & Lung	523	Chemo → Radi	52.0	Alive
IDC	66	M	45.0	GnP (7)	S-1	Lung → LN	469	Surgery → Chemo → Radi + Chemo	51.0	Dead
IDC	66	M	12.0	-	S-1	Lung	296.8	Surgery + Chemo	40.0	Dead
inv. IPMC	70	M	23.0	-	S-1	Lung	486	Surgery + Chemo	35.0	Dead
IDC	66	F	7.0	-	none	Para Ao LN	319.4	Radi + chemo	25.0	Dead
IDC	72	F	5.0	-	none	local	161.3	Radi	8.0	Dead
ACC	77	M	43.0	-	none	Para Ao LN	17.4	Radi → Chemo + Radi	42.0	Dead

*RFS* Recurrence-free survival, SAR: Survival after recurrence, GnP: Gemcitabine + nab-paclitaxel, *LN* Lymph node, *Chemo* Chemotherapy, *Radi* Radiotherapy, *IDC* Invasive ductal carcinoma, *inv*, *IPMC* Invasive intraductal papillary mucinous carcinoma, *ACC* Acinar cell carcinoma, *MCC* Mucinous cystadenocarcinoma

The reasons for LT was not being performed in the other 19 patients with OM were as follows: poor general condition, 5; patients’ refusal, 7; lost to follow, 4; technical difficulty performing radiation due to tumor location, 2; death due to heart failure, 1. Sixteen patients received chemotherapy and 3 patients did not. The median disease-free survival (DFI) of the 10 patients with LT and 19 patients without LT was 12 (range, 5–45) and 13 (range, 4–50) months, respectively (*p* = 0.7191). The median serum CA19-9 level at the time of recurrence in each group was 161.3 (range, 5.8–523) and 182.4 (range, 0.7–2145) U/mL, respectively (*p* = 0.9451).

### Survival analyses

**Fig. 2:**
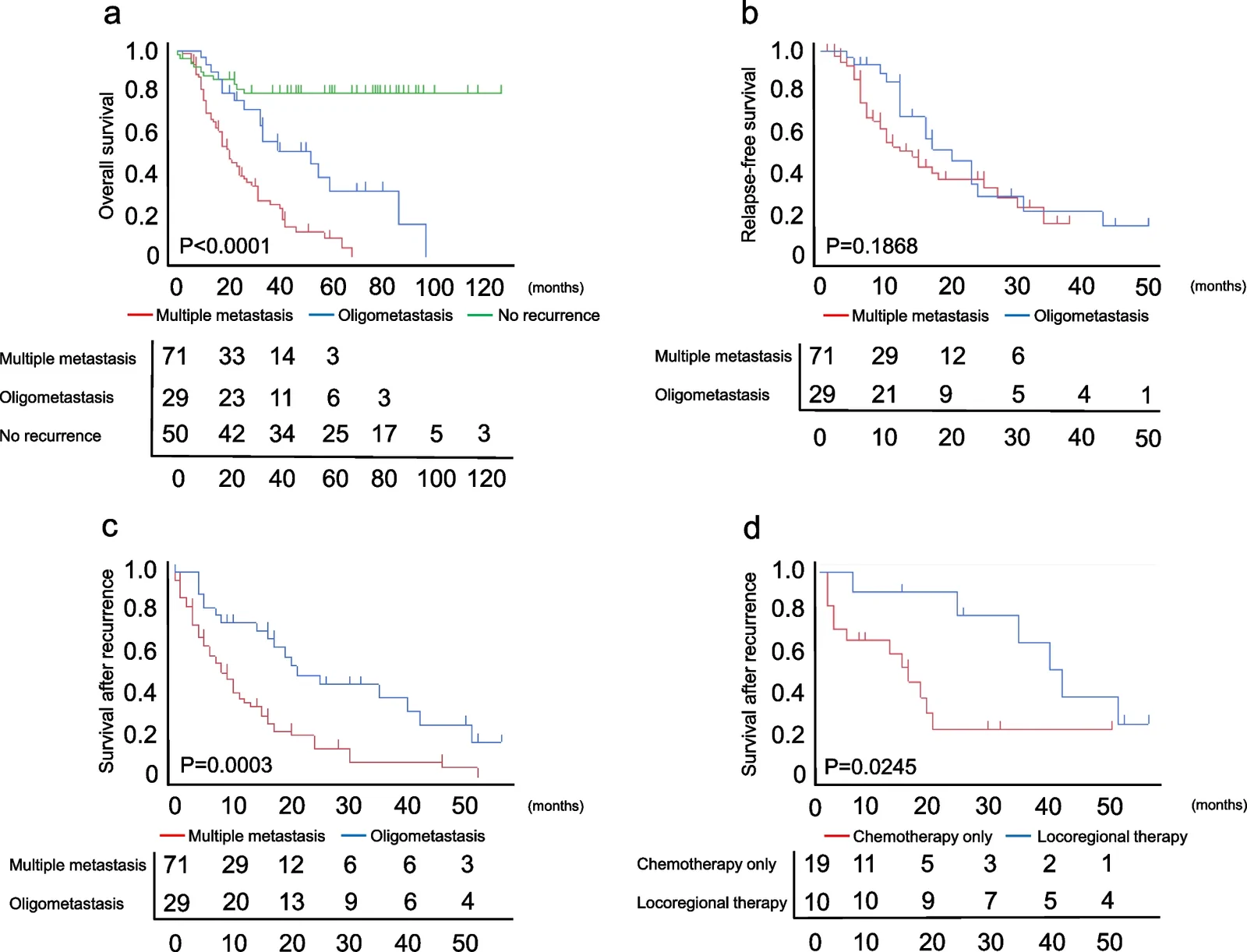
Survival analyses after surgical resection for pancreatic cancer. **A** Overall survival according to recurrence status: no recurrence, oligometastasis (OM), or multiple metastases (MM). **B** Disease-free interval in patients with OM and MM. **C** Survival after recurrence in patients with OM and MM. **D** Survival after recurrence in patients with OM according to treatment: locoregional therapy (LT) with or without chemotherapy versus chemotherapy alone. Patients with MM showed significantly worse survival than those with OM (A: *P* < 0.0001; C: *P* = 0.0003). Among patients with OM, those who underwent LT showed significantly longer survival after recurrence than those treated with chemotherapy alone (*P* = 0.0245)

The median postoperative follow-up period was 29.0 months, ranging from 0 to 126 months. There was a significant difference in overall survival (OS) among patients without recurrence, with OM, and with MM, with median OS of 85, 52, and 20 months, respectively (p < 0.0001) (Fig. 2a). No significant difference was observed in DFI between the OM (median, 14 months) and MM (median, 20 months, *p* = 0.1868) groups (Fig. 2b). However, there was a significant difference in survival after recurrence (SAR) between the two groups (median, 21 vs. 9 months, *p* = 0.0003) (Fig. 2c). Among patients with OM, those who underwent LT exhibited significantly better SAR (median, 42 months) compared with those who did not (median, 17 months, *p* = 0.0245) (Fig. 2d).

The clinical courses of patients who underwent LT are summarized in Table 3. Among the 10 patients treated with LT, five achieved survival after recurrence of more than 36 months. These long-term survivors included patients with isolated lung metastasis and those with para-aortic lymph node recurrence who received systemic chemotherapy combined with LT. Surgical resection was mainly performed for solitary pulmonary lesions that were considered resectable with acceptable invasiveness, whereas radiotherapy was mainly selected for local recurrence or para-aortic lymph node metastasis.

## Discussion

In this study, we demonstrated that the prognosis of patients with OM after surgery was better than that of patients with MM after surgery for PC. Furthermore, among patients with OM following surgery, those who underwent LT showed a better prognosis compared with those who received only chemotherapy. Additionally, combining LT for patients showing a response to chemotherapy may improve their prognosis.

The benefit of LT including radiation and surgical resection for OM after surgical resection of PC has rarely been evaluated. Studies by Schwartz et al. [14] and Groot et al. [15] reported that PC patients who underwent local resection for OM in the liver or lung showed better prognoses. Additionally, some studies highlighted non-surgical LT options, such as RFA (radiofrequency ablation) or TACE (transcatheter arterial chemoembolization), as beneficial treatments for OM after surgery for PC. [18, 19] These findings support our conclusion that LT, regardless of the type of treatment, is a feasible strategy to prolong the survival of patients with OM after surgery. Furthermore, our results suggest that the prognosis may be further improved by adding LT for OM patients with PC who receive chemotherapy.

The prognosis in the OM group was more favorable compared with that in the MM group. Although various studies have identified prognostic factors associated with better outcomes following OM, [10, 11, 20] the definition of OM in PC has not been standardized [21]. In these reports, OM was defined as limited disease with the presence of ≤ 4 metastases in the liver or lung with a CA 19–9 baseline < 1,000 U/mL and controlled disease after first-line chemotherapy [10], showing metastasis to a single organ, few countable lesions, and also a low serum CA 19–9 level [11]. Although our definition of OM did not include the CA19-9 level and the value at the time of recurrence was comparable between OM and MM groups in our series, some previous studies have included biological factors such as CA19-9 when defining OM. In contrast, our definition of OM was based mainly on the number and distribution of recurrent lesions and did not include CA19-9. Because CA19-9 did not clearly distinguish patients who underwent LT from those who did not in this cohort, its role in defining postoperative OM remains unclear and should be examined in future studies.

In addition to biological markers, the site of recurrence may also influence the clinical meaning of postoperative OM. Isolated pulmonary recurrence may represent a more indolent disease course than other recurrence patterns, whereas local recurrence or para-aortic lymph node recurrence may have different clinical implications. Therefore, the favorable outcomes observed in some patients in the present study may have been influenced by biologically favorable recurrence patterns, such as isolated lung metastasis, rather than by LT alone.

In patients with postoperative OM, we consider systemic chemotherapy before LT to be important for evaluating disease biology. Disease control during chemotherapy may help identify patients with relatively indolent recurrence who could be considered for LT. Therefore, upfront surgery should not be regarded as a routine strategy for postoperative OM. However, in selected patients with solitary pulmonary lesions, surgical resection may be considered for both diagnostic and therapeutic purposes when complete removal is feasible with acceptable invasiveness. Previous studies have also suggested that disease control after first-line chemotherapy and limited metastatic burden may be important factors in defining OM and selecting candidates for local therapy. Nevertheless, this strategy introduces selection bias because patients with disease control during chemotherapy are expected to have better outcomes. Therefore, the longer survival observed in the LT group should be interpreted cautiously. Modern combination chemotherapy regimens, such as FOLFIRINOX [22] and gemcitabine plus nab-paclitaxel [23], have improved outcomes in patients with metastatic pancreatic cancer. However, evidence remains limited regarding whether these regimens improve survival specifically in patients undergoing LT for postoperative OM. Therefore, the role of intensive systemic chemotherapy in this setting should be evaluated in future studies.

This study has several limitations. First, the study was retrospective and conducted at a single institution, and only 10 patients with postoperative OM underwent LT. Thus, our findings should be regarded as exploratory. Second, the decision to perform LT was made after multidisciplinary assessment, which may have introduced substantial selection bias. Several patients in the non-LT group did not undergo LT because of poor general condition, loss to follow-up, technical difficulty due to tumor location, or death from other causes. Therefore, the poorer survival observed in the non-LT group may partly reflect unfavorable clinical conditions or disease biology rather than the absence of LT itself. Third, the LT cohort was heterogeneous in terms of pathological type, metastatic site, and treatment modality. In particular, the inclusion of different recurrence sites and the use of both surgical resection and radiotherapy may have influenced the survival outcomes. Because postoperative OM in pancreatic cancer remains a heterogeneous and insufficiently defined condition, larger multicenter studies using more consistent selection criteria are needed to clarify the clinical role of LT.

## Conclusions

Patients with postoperative OM after pancreatic cancer surgery showed longer survival after recurrence than those with MM. In carefully selected patients with postoperative OM, LT combined with chemotherapy was associated with favorable survival outcomes. However, given the retrospective single-center design, small number of treated patients, and potential selection bias, LT should be viewed as a possible treatment option rather than an established therapeutic strategy. Further multicenter studies are warranted to clarify the clinical value of LT in this setting.

## Data Availability

The datasets used and/or analyzed during the current study are available from the corresponding author on reasonable request.

## Abbreviations

PC: Pancreatic cancer
OM: Oligometastasis
MM: Multiple metastases
LT: Locoregional therapy
UICC: Union for International Cancer Control
CT: Computed tomography
PET/CT: Fluorodeoxyglucose positron emission tomography
MRI: Magnetic resonance imaging
IDC: Invasive ductal adenocarcinoma
inv. IPMC: Invasive intraductal papillary mucinous carcinoma
ACC: Acinar cell carcinoma
MCC: Invasive mucinous cystadenocarcinoma
DFI: Disease-free survival
OS: Overall survival
SAR: Survival after recurrence
RFA: Radiofrequency ablation
TACE: Transcatheter arterial chemoembolization
